# On the Necessity of Artistic Knowledge and Critical Taste to Highest Success in the Dental Profession

**Published:** 1869-05

**Authors:** G. H. Perine


### SELECTED ARTICLES.

### ARTICLE VI.

### *On the Necessity of Artistic Knowledge and Critical Taste to Highest Success in the Dental Profession.*

By G. H. Perine, D.D.S.

The art of healing, including medicine and surgery, of which latter dentistry is a special department, centres upon itself a wider range of collateral science than any other. It draws from every source of information something which can be applied to the alleviation of the miseries of mankind. Dentistry being only one department of the art, does not necessarily demand from its professors so wide a range of learning as medicine and surgery combined, but there are doubtless few who practice it that are as yet aware how far the resources of the profession can be enlarged, by knowledge of principles and facts pertaining specially to other arts and professions. The object of this paper is to call the attention of the profession, especially its younger members, to the importance of the study of the fine arts, particularly that of portrait painting and modelling, with reference to the direct application of the knowledge and critical taste thus gained, to the practice of dentistry; and also to show how such application can be made to the rational correction of malformations and artificial deformities.

Comparatively few are gifted by nature with perfectly formed jaws and teeth, but in the present state of the art we should hesitate to avow that any ordinary case of malformation could not be corrected, and that without the sacrifice of teeth or the infliction of serious pain or inconvenience to the patient.

But natural malformations are scarcely more frequent than artificial ones caused by the injudicious and unnecessary extraction of teeth. It should be admitted as an axiom of modern dentistry, that *the extraction of any tooth from a young or old jaw, is certain to give rise to more or less permanent deformity.* Surely it is unnecessary at this day to substantiate this truth by argument. Every dentist has the proof at hand in the casts of jaws from which teeth have been removed. Let him compare the side of the jaw from which teeth have been taken with that in which the teeth remain, and assign if possible, any other reason for the difference, which is certain to be found.

Such deformity is much more likely to occur, and to assume exagerated proportions in young jaws, yet it is the constant practice of many otherwise excellent practitioners, to remove deciduous teeth, as though they were of no great consequence, thereby assuredly inflicting a lifelong injury upon the features of the little patient, unless a subsequent treatment shall avail to correct the injury. The plea for the practice is the correction of malformations, as when the teeth are crowded, and are growing "all awry," to make room for the remaining ones. Without wishing or intending to be severe, I assert that it would be just as rational to remove one entire jaw to make room for the other, as to remove one or more teeth to give others room. Further on I shall describe a more rational practice; before doing so, however, I wish to show how artistic taste, and, if possible, manual skill in painting or modelling, will aid the dentist in correcting deformity.

In most cases in which the aid of the surgeon is invoked for the correction of deformity, a standard of comparison by which the amount of deformity can be determined, is at hand, in the corresponding opposite part. A few operations about the face are exceptions. In cases of talipes when both feet are involved, his aim is to equalize as far as possible both members. The dentist is without this standard in many cases of artificial distortion; the deformity on one side drawing out of their proper position the muscles of the face upon the other, so that no very accurate idea can be obtained in the ordinary mode of examination, of the real form of the features previous to the date of the defect. To remedy the defect so as to make the features *better*, should not be the limit of our ambition; we should endeavor while we have the matter in hand to so operate that the *best* expression shall be given to the features compatible with the character of those features upon which it is not our province to operate.

We are here working upon plastic material which we can mould and fix in any desired position; why then should we stop at anything less than perfection, if we are prepared to judge accurately what is perfection? It is my intention to confine myself in discussing this part of the subject to the importance of an application of the principles of art in the treatment of such cases as I have mentioned, not to write an essay upon art; yet I cannot forbear calling the attention of the younger members of our profession to the part which the lower features of the face perform in the general expression. A very slight distortion is sufficient to render an otherwise beautiful face, almost ugly, as an experiment with an ordinary card photograph will easily demonstrate. Especially is this the case with the female face, the lower portions of which cannot be concealed by beard, and to which any deformity is a serious calamity.

I need not add that a dentist skilful in the correction of such defects, secures to himself a practice which although it may tax his patience, is certainly remunerative.

The distortions arising from the loss of teeth are in some cases so great that a comparison of the features with photographs taken before their extraction, will often surprise even one accustomed to making such comparisons. The extraction of the cuspids in childhood alters the features more than the removal of any others, yet these teeth are often ruthlessly sacrificed, by practitioners from whom a more rational practice ought to be expected. I have in my possession a photograph of a young lady now 24 years of age, who about two years since had the right upper cuspid tooth extracted. I am now treating her with a view to the correction of a marked distortion resulting from the loss of that tooth ; a distortion so marked that it has been a source of great mortification to the patient. The face is drawn to the right and, what upon the evidence of the photograph alluded to were once remarkably well-formed and expressive features, have been most sadly, though I trust not irreparably marred.

In cases of this kind, a photograph of the patient taken previous to the loss of teeth, is an invaluable guide to the dentist in correcting the defect. But it often happens that such a guide cannot be obtained. When this happens his power of analysis, and his artistic taste and knowledge are taxed to determine as far as possible from those portions of the general contour which remain undeformed, what must have been the natural form and expression previous to the occurrence of the deformity. And I assert that with a rational method of treatment, and all other things being equal, success in this difficult department of the art of dentistry will be in proportion to the artistic taste and judgment of the practitioner.

In cases of this kind I have been uniformly successful without recourse to the extraction of teeth, and I now proceed to give as briefly as I can my method of treatment. I do not claim this method as entirely original with me, although I might claim to be the inventor of some of the details. I shall content myself however with a mere description of the mode of practice which I have found the best, leaving it to the profession to judge how far I ought to be credited with any of its feature.

In treating these cases, I begin with the upper jaw, and as the principles involved are the same for both the upper and lower jaw, the description of the process need not comprise the latter ; I first fit a rubber plate to the roof of the mouth in the usual manner, and insert in sockets formed upon the borders of this plate, pins of compressed hickory corresponding to each tooth which it is desired to assume a more outward position. As soon as these teeth have yielded to the pressure so that the pins are loosened I substitute for them others which renew the pressure until they have yielded as far as may be requisite.

While the above process is going on, I at the same time compel the teeth which stand too far out to fall into line, by the following means: In the centre of the rubber plate above described, are inserted small hooks of platinum. Over these hooks I loop a small rubber band, (the small elastic bands used for holding bundles of tickets, etc., together, and of which I keep a supply on hand answer the purpose perfectly) and also loop it over the tooth whose position I wish to alter. These bands are the best things I have ever used for the purpose, their elasticity, and their softness being strong points in their favor. They can be renewed as often as required by the patient, and can be worn without any serious inconvenience.

By the means described the teeth are expanded or drawn in until they stand as regular and even as desired. But at this stage of the treatment the axes of the teeth extended would all meet at the apex of a cone of which the cusps of the teeth form a portion of the perimeter of the base.

Occlusion between them and the lower teeth is only partial, or wholly obviated. How then shall the jaw be expanded so that the fangs shall be thrown out and the teeth be made to assume their normal relations? I have found no difficulty in accomplishing this by the following means.

I fit a new plate to the roof of the mouth, forming upon it artificial cusps corresponding to the teeth in the lower jaw; upon these cusps the pressure of the lower jaw is received in the mastication of food, and more or less at all times and transmitted to the arch of the plate. A general expansion of the bones and tissues is the result. The whole jaw is enlarged, and the work is complete.

I am aware that many will doubt that these simple means will accomplish so much, but let those that doubt remember that the bony structures are plastic in their nature; especially so in youth; and that this plasticity if ever lost, is retained until late in life

Let them make the experiment and convince themselves, it will require patient attention perhaps, for many weeks or months; much reasoning with over fond parents to keep the apparatus applied with sufficient constancy, to secure a good result; but with favorable conditions, the results need not be doubtful; nay, they may be as certainly relied upon as those of any other operation in modern dentistry.

I use rubber plate in preference to any other, because its effects upon the teeth are more harmless, and its rigidity is ample.

In conclusion I desire to urge upon the younger members of our profession a candid consideration of the value of art culture. Although in our own day but little may be accomplished, the time is coming when this department of our art will assume an importance little dreamed of by those who are content to tread in the old beaten path, and by whom any attempt at advancement, is regarded as an unwarantable innovation.—*Med. Gazette.*

